# Resilience to Plasma and Cerebrospinal Fluid Amyloid-β in Cognitively Normal Individuals: Findings From Two Cohort Studies

**DOI:** 10.3389/fnagi.2021.610755

**Published:** 2021-02-24

**Authors:** Li Lin, Yu Sun, Xiaoqi Wang, Li Su, Xiaoni Wang, Ying Han

**Affiliations:** ^1^Department of Neurology, XuanWu Hospital of Capital Medical University, Beijing, China; ^2^Department of Psychiatry, University of Cambridge, Cambridge, United Kingdom; ^3^Center of Alzheimer's Disease, Beijing Institute for Brain Disorders, Beijing, China; ^4^National Clinical Research Center for Geriatric Disorders, Beijing, China

**Keywords:** resilience, Alzheimer's disease, amyloid, cognitive decline, cognitively normal

## Abstract

**Objective:** To define resilience metrics for cognitive decline based on plasma and cerebrospinal fluid (CSF) amyloid-β (Aβ) and examine the demographic, genetic, and neuroimaging factors associated with interindividual differences among metrics of resilience and to demonstrate the ability of such metrics to predict the diagnostic conversion to mild cognitive impairment (MCI).

**Methods:** In this study, cognitively normal (CN) participants with Aβ-positive were included from the Sino Longitudinal Study on Cognitive Decline (SILCODE, *n* = 100) and Alzheimer's Disease Neuroimaging Initiative (ADNI, *n* = 144). Using a latent variable model of data, metrics of resilience [brain resilience (BR), cognitive resilience (CR), and global resilience (GR)] were defined based on the plasma Aβ and CSF Aβ. Linear regression analyses were applied to investigate the association between characteristics of individuals (age, sex, educational level, genetic, and neuroimaging factors) and their resilience. The plausibility of these metrics was tested using linear mixed-effects models and Cox regression models in longitudinal analyses. We also compared the effectiveness of these metrics with conventional metrics in predicting the clinical progression.

**Results:** Although individuals in the ADNI cohort were older (74.68 [5.65] vs. 65.38 [4.66], *p* < 0.001) and had higher educational levels (16.3 [2.6] vs. 12.6 [2.8], *p* < 0.001) than those in the SILCODE cohort, similar loadings between resilience and its indicators were found within both models. BR and GR were mainly associated with age, women, and brain volume in both cohorts. Prediction models showed that higher CR and GR were related to better cognitive performance, and specifically, all types of resilience to CSF Aβ could predict longitudinal cognitive decline.

**Conclusion:** Different phenotypes of resilience depending on cognition and brain volumes were associated with different factors. Such comprehensive resilience provided insight into the mechanisms of susceptibility for Alzheimer's disease (AD) at the individual level, and interindividual differences in resilience had the potential to predict the disease progression in CN people.

## Introduction

Alzheimer's disease (AD) was originally defined as a clinical pathologic disease mainly based on clinical symptoms and neuropathologic changes like deposition of amyloid-β (Aβ) and hyperphosphorylated tau. Recent developments of AD have proposed research frameworks based on biomarkers (McKhann et al., 2011; Jack et al., 2018), which enhanced the understanding of the mechanism and brought substantial research interest to the preclinical stage of AD (Dubois et al., 2018; Jessen et al., 2018; Slot et al., 2018; Li et al., 2019). However, studies have shown that biomarkers and cognitive performance were discordant in some individuals with the pathology of AD often due to individual variations in resilience to the pathology of AD (Katzman et al., 1988; Ghisays et al., 2020). Therefore, as a theoretical construct, terms like reserve, resilience, and maintenance were defined to enhance the understanding of the individual difference related to diseases of the brain (Stern et al., 2018). Resilience, which represents the degree of structural and cognitive deficits associated with the pathology of AD, can be divided into brain resilience (BR) and cognitive resilience (CR) (Rentz et al., 2017) or a combination of both [e.g., global resilience (GR); Hohman et al., 2016; Arenaza-Urquijo and Vemuri, 2018)]. High or low resilience reflects better or worse than predicted properties of the brain or cognitive performances based on the pathological burden. Compared with the traditional index of the reserve including educational level, intelligence quotient (IQ), and occupational complexity, resilience defined by residual approaches provided a feasible quantitative measure of the impact of pathology on cognition (Ewers, 2020).

Currently, studies have found that high resilience may slow the rate of cognitive decline (van Loenhoud et al., 2019; Ossenkoppele et al., 2020), and the protective effect was more notable in the early stage before the cognitive impairment (Lo et al., 2013; Arenaza-Urquijo et al., 2017), which had important implications for intervention in the cognitively normal (CN) stage. In addition, these studies generally used cerebrospinal fluid (CSF) Aβ and PET Aβ to measure the level of the amyloid deposition. However, both methods are invasive or expensive, which are not suitable for widespread screening in the preclinical phase of AD. The advent of plasma Aβ has provided an alternative that is affordable and less invasive. Previous studies have shown the relationship between plasma Aβ and central Aβ (Hanon et al., 2018; Chatterjee et al., 2019; Schindler et al., 2019; Vergallo et al., 2019). The biomarker of amyloid peptide and the Alzheimer's disease risk (BALTAZAR) study found that the plasma Aβ was associated with cognitive performance, apolipoprotein E (APOE)-ε4 status, and CSF Aβ in cross-sectional analyses (Hanon et al., 2018). In a longitudinal study, Schindler et al. found that the plasma Aβ was inversely correlated with the baseline PET Aβ and correlated with the baseline CSF Aβ in CN older adults and could be used to predict the future amyloid status (Schindler et al., 2019).

However, it is still unclear that, in the context of CN individuals, which factors contribute to these metrics of resilience (BR, CR, and GR) to Aβ and whether the levels of resilience defined by plasma Aβ and CSF Aβ could both help predict the baseline and longitudinal cognitive decline. To address the questions mentioned above, we, therefore, quantified the metrics of resilience based on plasma Aβ and CSF Aβ. Using linear regression analyses, we tested whether demographic (age, sex, and educational level), genetic (APOE-ε4), and imaging markers are associated with these metrics of resilience. Then for investigating the added predictive value of different metrics of resilience, we explored the hypothesis that these metrics of resilience could help predict the diagnostic conversion and high resilience could slow down the cognitive decline longitudinally in CN individuals.

## Methods

### Participants

Participant data from the Sino Longitudinal Study on Cognitive Decline (SILCODE) project (Li et al., 2019) from March 2017 to October 2018 and Alzheimer's Disease Neuroimaging Initiative (ADNI) (Weiner and Veitch, 2015) were used for this retrospective study. The SILCODE is a longitudinal observational project focusing on elderly Chinese people. Its primary goal was to identify the individuals in preclinical AD who would convert to mild cognitive impairment (MCI) and understand the disease mechanism. The ADNI was launched in 2003 as a public–private partnership whose goal was to test whether biological markers and clinical assessments could be combined to measure the progression of individuals in the spectrum of AD. The Aβ-positive CN participants were recruited from the SILCODE (*N* = 100) and the ADNI (*N* = 144). The median follow-up was 9.4 months [interquartile range (IQR): 0–17.3] for the SILCODE and 70.8 months (IQR: 35.9–91.2) for the ADNI. All CNs underwent clinical and neuropsychological assessments, plasma (SILCODE) or examination of the CSF (ADNI), MRI scans at baseline, and had a normal performance on neuropsychological tests adjusted for age, sex, and education. Participants with the following conditions were excluded: current major psychiatric diagnosis such as depression and anxiety, serious neurologic diseases, diseases that could cause cognitive decline (e.g., thyroid dysfunction, severe anemia, syphilis, or HIV), a history of brain lesions, or head traumas (additional inclusion/exclusion criteria can be found at www.adni-info.org and https://www.clinicaltrials.gov/ct2/show/study/NCT03370744).

### Standard Protocol Approvals, Registrations, and Patient Consent

Informed written consent was obtained from all participants at each site, and study procedures were approved by the institutional review board at each center. The ADNI and the SILCODE are listed in the ClinicalTrials.gov registry (ADNI-1: NCT00106899; ADNI-2: NCT01231971; ADNI-GO: NCT01078636; ADNI-3: NCT02854033; and SILCODE: NCT03370744).

### Neuropsychological Assessments

Across both studies, the neuropsychological assessment covered similar cognitive domains. To aid comparability and calculate the CR, we chose six neuropsychological measures used by both studies and combined them into three domains: memory, language, and executive. The six measures were the Boston Naming Test and the Animal Naming for the language domain, the Trail Making Test A and the Trail Making Test B for the executive domain, and the auditory verbal learning test (AVLT) delayed recall and the delayed recognition scores for the memory domain. Scores of the Mini–Mental State Examination (MMSE) and the Montreal Cognitive Assessment (MoCA) were used to assess global cognition. All test scores were z-transformed within each test to remove the bias among measures. Within each domain, z-transformed scores were averaged to obtain a composite score.

### MRI Acquisition and Processing

Structural MRI scans were acquired on 3T scanners from GE Healthcare (Chicago, USA), Philips Medical Systems (Eindhoven, Netherlands), and Siemens Medical Solutions (Erlangen, Germany) (http://adni.loni.usc.edu/methods/documents) in the ADNI study and acquired on 3T SIGNA PET/MR (GE Healthcare) and Tim Trio (Siemens Medical Solutions) in the SILCODE study. The mean interval between the baseline cognitive visit and related neuroimaging visit was 23 and 22 days for the SILCODE and the ADNI cohorts, respectively. Structural MR images were processed using SPM12 (http://www.fil.ion.ucl.ac.uk/spm/software/spm12). In the preprocessing, structural images were removed from the non-brain tissue; segmented into the gray matter (GM), the white matter (WM), and the CSF; and then modulated and normalized into the MNI template. A 6 mm full width at half maximum (FWHM) Gaussian isotropic kernel was used to spatially smooth the normalized images. The intracranial volume (ICV) was computed as the sum of the GM, the WM, and the CSF. The left and the right hippocampal were determined for subsequent analysis.

### Biomarker Collection and Analyses of the Plasma and the CSF

In the SILCODE cohort, plasma samples were collected in polypropylene tubes with ethylenediaminetetraacetic acid (EDTA). Samples were centrifugated, aliquoted, and stored at −80°C. Plasma samples were measured using the kit: Aβ Peptide Panel 1 (4G8) Kit (Mesoscale Diagnostics, Rockville, Maryland, USA), and they were randomized and measured in duplicate with the same aliquot, blinded for clinical diagnosis. Levels of Aβ42 showed good average coefficients of variation of duplicate measurements (4.14% CV) and within the detection limit (2.5–1,271 pg/ml). In the ADNI, lumbar puncture was performed as previously described (http://www.adni-info.org/). Values of Aβ42 in samples of the CSF were generated by a novel, fully automated electrochemiluminescence immunoassay (Elecsys assay) (Bittner et al., 2016) and downloaded from the LONI site (provided in UPENNBIOMK9.csv and UPENNBIOMK10.csv files).

### Gaussian Mixture Modeling

Since there is no accepted standard cut-point to determine amyloid status in relation to plasma Aβ, we calculated the cut-point to determine the amyloid status with the Gaussian mixture modeling in the SILCODE cohort. This approach was suitable since it is data-driven and does not assume similar distributions of levels of Aβ across cohorts (Mormino et al., 2014; Wang et al., 2016). First, 1–5 Gaussian distributions allowing for either equal or unequal variances were fit to the data, and the number of distributions that best described the data was determined by the Bayesian information criterion (BIC) (see Supplementary Figure 1). We found that the optimal model contained two Gaussian distributions that reflected abnormal vs. normal Aβ. Each individual was assigned a probability of belonging to either the abnormal or normal distribution. Considering the relatively high false positive rate of abnormal Aβ based on only CN population and plasma Aβ, CNs with >50% probability of belonging to abnormal Aβ distribution as well as smaller than 25% uncertainty corresponding to the classification were labeled Aβ+. Otherwise, they were labeled Aβ-. The final cut-points were 11.43 pg/ml in the SILCODE group. For the ADNI group, we used a pre-defined cut-point (980 pg/ml) to determine the amyloid status (Hansson et al., 2018).

### Statistical Analyses

**Figure 1:**
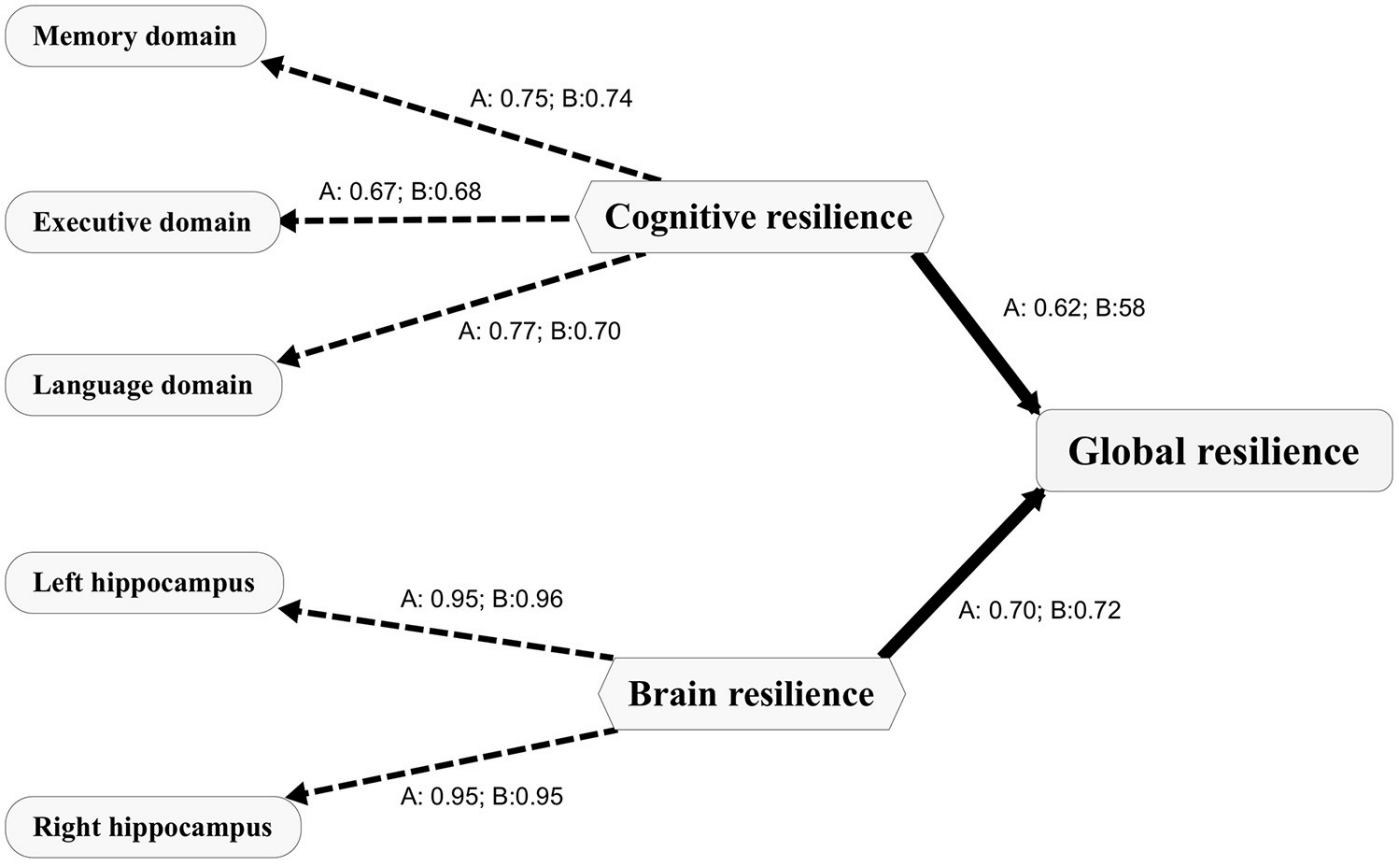
Path diagram depicting the PLS models for both cohorts. The PLS path model includes the outer model and the inner model. The outer model includes indicators presented as capsules and the first order latent variables presented as hexagons. The inner model depicts the relationships between the first order latent variables (CR and BR) and the second order latent variable (GR). The loadings between a latent variable and its indicators are presented as numbers. A, the PLS model for the ADNI; B, the PLS model for the SILCODE. CR, cognitive resilience; BR, brain resilience; GR, global resilience. PLS, partial least squares; ADNI, Alzheimer's Disease Neuroimaging Initiative; SILCODE, Sino Longitudinal Study on Cognitive Decline.

Statistical analyses were performed in the R version 3.6.2 (http://www.r-project.org/) and R-package “plspm” was used to construct the partial least squares (PLS) path models. Two multivariate PLS path models (Figure 1) were constructed to quantify the BR and CR and a second-order latent composite measure (GR) for each cohort. The PLS path model, which is for studying complex multivariate relationships among observed and latent variables, is the partial least squares approach to the structural equation modeling. It provides a framework for analyzing multiple relationships between a set of blocks of variables. A full path model comprises two submodels: an outer model that reflects the relationships between each latent variable and its block of indicators and an inner model that reflects the relationships between latent variables. In this study, for the outer model, memory, language, and executive domain residuals were indicators of the CR when left and right hippocampus volume residuals were indicators of the BR. For the inner model, the GR was derived from its latent variables (CR and BR). Specifically, we performed individual linear regression models between composite scores/hippocampal volumes and plasma/CSF Aβ levels and used the standardized residual as an indicator of CR/BR. For example, memory domain residuals to Aβ were calculated as residuals from a regression model with memory domain scores as the outcome and Aβ as the predictor, representing as one of the indicators of the CR.

To evaluate which factors contribute to resilience, we performed two sets of multivariable linear regression models. In the first set, multivariable linear regression models were performed with age, sex, educational levels, APOE-ε4 status, and the whole brain volume as independent variables and the respective metric of resilience as a dependent variable. In the second set, the cognitive scores and brain volumes for residuals calculation were also included as independent variables. Then, linear mixed effect models were used for longitudinal cognitive analyses. The global cognition test (MoCA) was used as the outcome variable, and fixed effects included our interested effects (i.e., the effect of resilience of interest * time) as well as other effects that we wanted to test or control, including time (from the baseline), age, sex, education, and APOE-ε4 status. The random effect included the intercept of an individual. In addition to the original mixed effect models, we also performed another set of mixed effect models adjusting the effects of the original cognitive and brain variables used to derive resilience and included the time interactions with each of these variables. To further evaluate the utility of resilience in predicting the conversion from normal controls (NCs) to MCI, the Cox analysis for each metric of resilience was performed. For visualization purposes, we dichotomized the sample according to low vs. high resilience (CR, BR, or GR) using a median split. Using these dichotomized groups, we computed three 2-level variables (CR: CR+/CR–; BR: BR+/BR–; and GR: GR+/GR–) and Kaplan–Meier survival curves were created to show the conversion rate from the baseline with respect to resilience. Finally, for evaluating the added value of resilience for predicting the longitudinal cognitive decline, we used the Akaike information criterion (AIC) and the BIC to evaluate both the predictive models with conventional indicators (MoCA/MMSE and hippocampal volume/brain volume) and the predictive models with conventional indicators plus resilience. A lower AIC or BIC value indicates a better model. The level of significance was set at *p* < 0.05.

## Results

### Characteristics of Participants

A total of 244 participants were included in the study. From the SILCODE group, 100 participants were included [59 (59%) female; mean (SD) age, 65.38 (4.66); mean (SD) MMSE score, 28.84 (1.13)]. From ADNI group, 144 participants were included [74 (51.4%) female; mean (SD) age, 74.68 (5.65); mean (SD) MMSE score, 29.08 (1.10)]. The characteristics and clinical details of the participants are presented in Table 1. The average age [65.38 (4.66) vs. 74.68 (5.65), *p* < 0.001] and educational level [12.6 (2.8) vs. 16.3 (2.6), *p* < 0.001] of participants in the SILCODE were significantly lower than those in the ADNI.

**Table 1:** Characteristics of the participants of the study.

	**SILCODE**	**ADNI**	***P***
Sample size, *n*	100	144	
Age	65.38 (4.66)	74.68 (5.65)	<0.001
Female (%)	59 (59)	74 (51.4)	0.297
Education level	12.6 (2.8)	16.3 (2.6)	<0.001
MMSE	28.84 (1.13)	29.08 (1.1)	0.094
MoCA	25.73 (2.08)	25.63 (2.3)	0.752
AVLT-delay^a^	7.03 (1.85)	7.52 (3.83)	/
AVLT-recog^a^	22.46 (1.41)	12.72 (2.61)	/
AFT	19.13 (4.54)	20.88 (5.22)	0.007
BNT	24.96 (2.86)	28.03 (2.27)	<0.001
STT-A^b^	59.95 (17.48)	35.28 (13)	/
STT-B^b^	138.64 (35.73)	88.86 (44.31)	/
APOE-ε4 (%)	21 (21)	61 (42.4)	0.001
Aβ level^c^	8.9 (1.87)	808.18 (281.32)	/

*MMSE, Mini–Mental State Examination; MoCA, Montreal Cognitive Assessment; AVLT-delay, auditory verbal learning test-delay recall; AVLT-recog, auditory verbal learning test-recognition; STTA, shape trail making test A; STTB, shape trail making test B; AFT, animal fluency test; BNT, Boston Naming Test; APOE, apolipoprotein E. Data are presented as mean (SD) unless otherwise indicated*.

a *AVLT in the Sino Longitudinal Study on Cognitive Decline (SILCODE) was HuaShan version and in the Alzheimer's Disease Neuroimaging Initiative (ADNI) was Rey AVLT version A/B*.

b *STT-A and STT-B in the SILCODE were Alternative Shape Trail Making Test A and B and in the ADNI were Shape Trail Making Test A and B*.

c *Source of amyloid-β (Aβ) in the SILCODE was the plasma and in the ADNI was the cerebrospinal fluid (CSF)*.

### The PLS Path Model

The PLS path models for the SILCODE and the ADNI cohorts are presented in Figure 1. For evaluating the quality of the two models, we assessed three aspects of the measures of the model. Both models showed good one-dimensionality of the indicators (SILCODE: mean Cronbach's alpha = 0.67, mean Dillon–Goldstein's rho = 0.81, mean first eigenvalue = 1.79; ADNI: mean Cronbach's alpha = 0.69, mean Dillon–Goldstein's rho = 0.82, mean first eigenvalue = 1.80). Furthermore, the indicators were well-explained by its latent variable as average loading for the CR and the BR was 0.81 for the SILCODE cohort and 0.82 for the ADNI cohort. The degree to which a given construct was different from other constructs was good (all cross-loadings in two models <0.26). In sum, both models fit well and had a goodness-of-fit score of 0.73.

### Factors Associated With Resilience

Multivariable models (Table 2) showed that factors contributing to different metrics of resilience were overall consistent in both cohorts. Younger age was associated with higher levels of all three metrics of resilience. Specifically, higher educational levels were related with greater CR and larger whole brain volume was related to greater BR and GR. After including the original cognitive and brain measures that resilience derived from Supplementary Table 1 the young, women, and large brain volume were associated with the BR and the GR while the CR was only related to cognitive composite scores.

**Table 2:** Demographic, genetic, and neuroimaging factors associated with metrics of resilience to Aβ.

**Variables**	**Cognitive resilience**		**Brain resilience**		**Global resilience**	
	**Standardized β**	***P***	**Standardized β**	***P***	**Standardized β**	**P**
**SILCODE**						
Age	−0.207	0.027	−0.217	0.033	−0.277	0.005
Sex	−0.240	0.021	−0.090	0.416	−0.203	0.06
Educational levels	0.362	<0.001	−0.079	0.436	0.155	0.112
APOE-ε4	0.143	0.129	0.002	0.985	0.082	0.398
Whole brain volume	0.161	0.126	0.248	0.032	0.271	0.015
**ADNI**						
Age	−0.284	0.001	−0.332	<0.001	−0.409	<0.001
Sex	−0.093	0.322	−0.026	0.769	−0.075	0.372
Educational levels	0.166	0.047	−0.010	0.903	0.096	0.198
APOE-ε4	0.010	0.904	−0.012	0.872	−0.003	0.97
Whole brain volume	0.110	0.241	0.300	0.001	0.279	0.001

### Longitudinal Cognitive Progress

**Figure 2:**
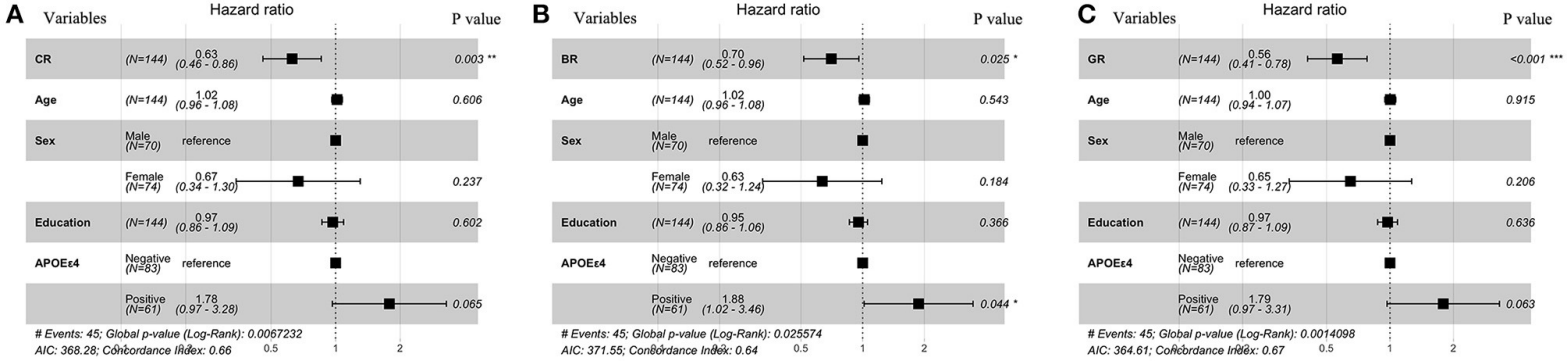
Forest plots for metrics of resilience as predictive factors for clinical conversion. Resulting hazard ratios for models of cox regression classified by **(A)** CR, **(B)** BR, and **(C)** GR. All covariates were continuous except for sex and APOE status which were dichotomous. CR, cognitive resilience; BR, brain resilience; GR, global resilience.

**Figure 3:**
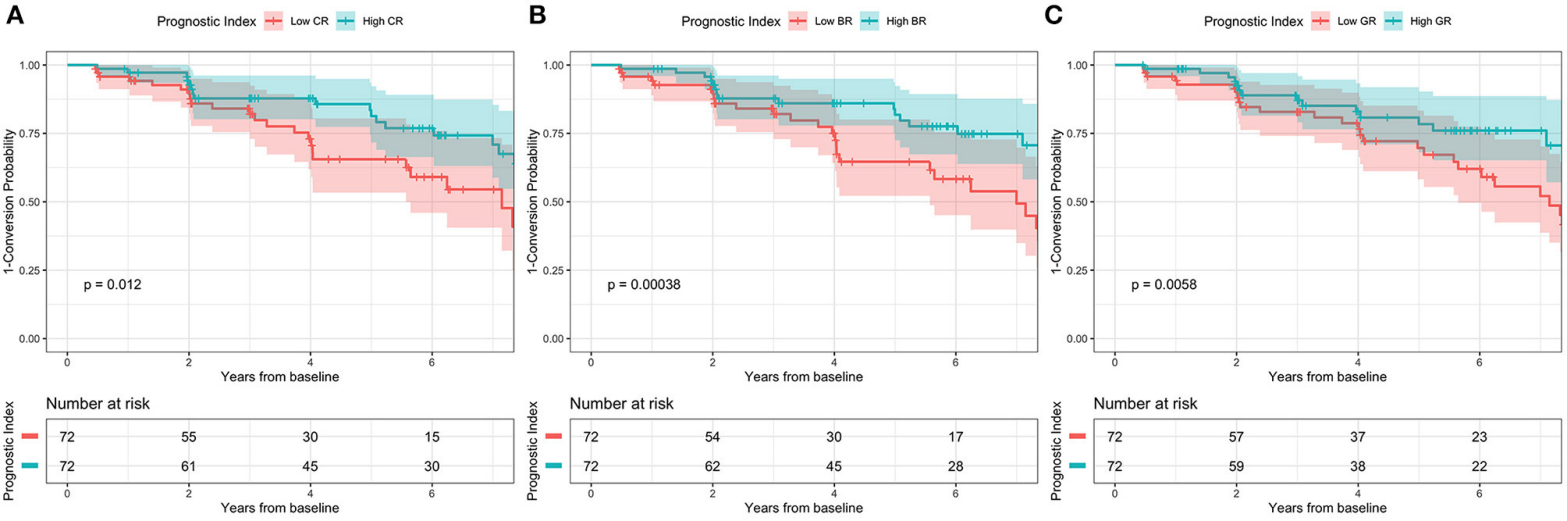
Kaplan-Meier survival curve according to 2-level variable in the ADNI cohort. High conversion rate was associated with **(A)** low CR, **(B)** low BR, and **(C)** low GR. CR, cognitive resilience; BR, brain resilience; GR, global resilience.

Linear mixed models (Table 3) in two cohorts both showed that higher CR (SILCODE: β = 1.064, *p* < 0.001; ADNI: β = 0.814, *p* = 0.002) and higher GR (SILCODE: β = 0.677, *p* < 0.001; ADNI: β = 0.661, *p* = 0.007) were associated with better cognitive performances. We also identified a significant positive interaction between all metrics of resilience and time in the ADNI cohort and a similar trend in the SILCODE cohort, suggesting that participants with a higher level of resilience would perform better over time. After controlling the baseline cognitive performance and hippocampal volumes (Supplementary Table 2), associations between resilience and cognition were insignificant in the SILCODE cohort, whereas higher CR (β = 0.977, *p* = 0.004), BR (β = 0.565, *p* < 0.001), and GR (β = 0.420, *p* = 0.036) in the ADNI cohort continued to predict slower cognitive decline in the CNs. As the results of Cox regression models and survival curves showed (Figures 2, 3), all three metrics of resilience were protective factors to clinical progression (all values of *p* < 0.05). Information criteria for prediction models with and without resilience were provided in Table 4. Compared with the predictive models with conventional variables (MMSE and brain volume), models with both conventional variables and resilience showed smaller AIC and BIC values (CR: AICΔ = −4.64, BICΔ = −2.84; BR: AICΔ = −5.56, BICΔ = −3.76; GR: AICΔ = −12.15, BICΔ = −10.34). Similar results were found in the comparison of the predictive models with another combination of conventional variables and the predictive models with conventional variables and resilience (Supplementary Table 3).

**Table 3:** Results of the mixed effect models to predict global cognition in both cohorts.

**Main effects**	**SILCODE**			**ADNI**		
	**β**	***t***	***P***	**β**	***t***	***P***
CR	1.064	5.811	<0.001	0.814	3.171	0.002
CR * years	−0.058	−0.302	0.763	0.118	3.069	0.002
BR	0.163	0.838	0.403	0.035	0.137	0.891
BR * years	0.146	0.841	0.402	0.197	5.302	<0.001
GR	0.677	3.63	<0.001	0.661	2.706	0.007
GR * years	0.058	0.328	0.744	0.217	5.972	<0.001

**Table 4:** Information criteria for prediction models with and without resilience.

**Model**	**AIC**	**Δ**	**BIC**	**Δ**
**Cognitive predictor^a^**				
Without cognitive resilience	374.86	–	383.89	–
With cognitive resilience	370.21	−4.64	381.05	−2.84
**Brain predictor^b^**				
Without brain resilience	372.97	–	382.01	–
With brain resilience	367.41	−5.56	378.25	−3.76
**Combined^c^**				
Without global resilience	373.15	–	383.99	–
With global resilience	361.00	−12.15	373.65	−10.34

*AIC, Akaike information criterion; BIC, Bayesian information criterion. All models were corrected for age, sex, educational levels, and APOE-ε4 status*.

a *This model included MMSE as the predictor*.

b *This model included brain volume as the predictor*.

c *This model included both MMSE and brain volume as the predictor*.

## Discussion

This study provides information about different phenotypes of resilience, their associations with demographic, genetic, and neuroimaging factors in a relatively large number of CNs. Two latent variable models to quantify the metrics of resilience related to Aβ in two cohorts were constructed. Results from this study suggested that younger individuals, women, and people with larger brain volumes were related to greater brain and GR when exposed to the Aβ burden. All three phenotypes of resilience based on the plasma and the CSF Aβ were observed to have a protective effect against cognitive decline in long-term follow-up. These metrics of resilience may capture additional information when the consequence of the clinical progression can be predicted than conventional cognitive and brain indicators.

Metrics of resilience were defined with the PLS model separately in each cohort and the loadings between cognitive indicators, brain indicators, and the respective resilience as well as the loadings between the BR, the CR, and the GR were calculated. The framework and goodness-of-fit in both models were similar and consistent with the previous work, indicating to a certain extent that the residualization approach was a feasible and reliable method of quantifying the metrics of resilience. Previous literature (Jagust, 2013; Scholl et al., 2016; Jack et al., 2019) had suggested that the brains were more vulnerable to various kinds of damages in aging and the prevalence of pathologic biomarkers in AD were found to be different in women and men with respect to age. Our results had confirmed the associations of these factors and resilience and indicated that age, sex, and brain volume were important for investigating the BR and the GR. In CN individuals, women, young individuals, or larger brain volume sustained better cognitive function and the preservation of the brain structure when exposed to pathological changes. Explanations could be the neural compensation mechanism, the protective effects of sex steroid hormones before menopause, the gene expression of heterochrony, and the threshold models of brain reserve (Satz, 1993; Berchtold et al., 2008; Pike et al., 2009). Intriguingly, women displayed both higher levels of pathologic biomarkers in AD and higher resilience (Jack et al., 2019), which seems to be congruent. However, the protective effect of resilience may diminish with age and the accumulation of pathological changes. In post-menopausal women, the depletion of sexual hormones, the gene expression changes in specific brain regions (like superior frontal gyrus), and high Aβ burden accumulation would accelerate cognitive decline although they remain clinically normal (Yuan et al., 2012; Jack et al., 2017). Compared with factors associated with the BR and the GR, we found a specific association between educational levels and CR as CN individuals with higher education level could cope with more severe pathological burden (Kemppainen et al., 2008; Pettigrew et al., 2017; van Loenhoud et al., 2019). The explanation of how education enhanced the reserve capacity and slowed down cognitive decline in individuals with accumulated pathological deposits was still unclear and was even challenged since the paradoxical results of education on modifying longitudinal cognitive decline (van Loenhoud et al., 2019; Wilson et al., 2019) though it was generally accepted that higher educational levels before progressing into the cognitive impaired stage were beneficial to our brains. However, after controlling the cognitive performance, the association between the CR and education levels disappeared, suggesting this relation was poor and more likely reflected the association between the baseline cognition and educational levels.

We found no association between the APOE genotype and the resilience at baseline in both cohorts. The dissociation of the APOE phenotype and the resilience could be caused by the dual effects on the brain of different APOE genotypes and the effect of the APOE on pathology was dominated in the later stage in AD. Consistent with this explanation, Wolk et al. (2010) reported APOE-ε4 carriers with AD displayed severe impairments in the memory domain rather than in execution and language domains. Another report (Pievani et al., 2009) found carriers were associated with greater temporal volume loss, and non-carriers were associated with greater frontoparietal volume loss.

Although previous studies exploring resilience have shown that higher resilience scores were associated with better cognitive performances and a smaller risk of the clinical progression, many of them defined the resilience based on educational levels, reading abilities, or lifestyle (Roe et al., 2008; Kaup et al., 2015; Vemuri et al., 2017). These metrics remained stable in the elderly and hardly reflected the current level of resilience of an individual. Therefore, they may fail to reflect the individual difference in cognitive or structural brain processes over time. However, several investigators have found residual approaches based on cognitive/brain predictors and biomarkers in AD to quantify that resilience could provide more information at the individual level over time and include information from various cognitive domains and biomarkers (Reed et al., 2010; Hohman et al., 2016). With this approach, we found that all types of resilience could predict longitudinal global cognitive changes, even adjusting for the baseline cognitive performance and hippocampal volumes and considering conventional predictors. This finding suggested that resilience was not only associated with demographic, genetic, cognitive, and imaging features but also provided added information about the prognosis.

The strength of our study lies in a relatively large sample of Aβ-positive CN individuals with demographic, genetic, neuroimaging, and biomarker data of AD, and a longitudinal design allowed us to inform the impact of different resilience to the clinical progression. Two cohorts provided the possibility of validating factors contributing to the different metrics of resilience and the effects of resilience on cognitive decline.

The present study has several limitations. The differences in characteristics of the participants and measurements across two cohorts led to only comparable cognitive tests with respect to the included three domains. This limitation was partly resolved by the combination of test scores and transformation. Another limitation was the different ranges of follow-up and different methods of accessing the amyloid in the two cohorts. The short follow-up period for the SILCODE cohort not only resulted in the absence of the Cox regression analysis for this cohort, as there were no sufficient clinical progression datapoints available for analysis but also may lead to the relatively poor predictive value of resilience compared to that in the ADNI cohort, though growing evidence has demonstrated that plasma Aβ concentrations are highly correlated with brain amyloidosis (Nakamura et al., 2018; Risacher et al., 2019). In addition, as the false positive rate for the classification of the Aβ status if only based on the CSF or the plasma was higher than those based on multiple methods or Aβ PET, we, therefore, adjusted the cut-point within the SILCODE by controlling the classification uncertainty of the Aβ abnormal below 25% and used a pre-defined cut-point in the ADNI that was determined to optimize the concordance with the Aβ PET visual read.

## Conclusion

This study provides information about the associations between the resilience based on the plasma Aβ and the CSF Aβ and demographic, genetic, and neuroimaging factors in Aβ-positive CN individuals. We found that younger individuals, women, and people with larger brain volumes were associated with higher brain and GR. Metrics of resilience based on Aβ had a protective effect against the clinical progression and could provide additional information beyond the cognitive performance and imaging features in CN people.

## Data Availability Statement

The data of SILCODE used and/or analyzed during the current study are available from the corresponding author on reasonable request. The data of ADNI can be found at the the Alzheimer's Disease Neuroimaging Initiative website (adni.loni.usc.edu).

## Ethics Statement

The studies involving human participants were reviewed and approved by XuanWu Hospital of Capital Medical University and the institutional review board at each center of ADNI. Informed written consent was obtained from all participants.

## Author Contributions

LL and YH contributed conception and design of the study. LL, YS, XiaonW, XiaoqW, and YH did the clinical assessments and data acquisition. LL, YS, XiaonW, and LS did the data analysis and interpretation. All authors contributed to manuscript revision, read and approved the submitted version.

## Conflict of Interest

The authors declare that the research was conducted in the absence of any commercial or financial relationships that could be construed as a potential conflict of interest.
