# Supplementary material for: Resilience to Plasma and Cerebrospinal Fluid Amyloid-β in Cognitively Normal Individuals: Findings From Two Cohort Studies
Source: Front Aging Neurosci. 2021 Feb 24;13:610755. doi: 10.3389/fnagi.2021.610755 (PMC7943465; doi:10.3389/fnagi.2021.610755)
Supplement: Supplementary file 1 [file Table_1.DOCX]

Supplementary Material

# Supplementary Figures

#
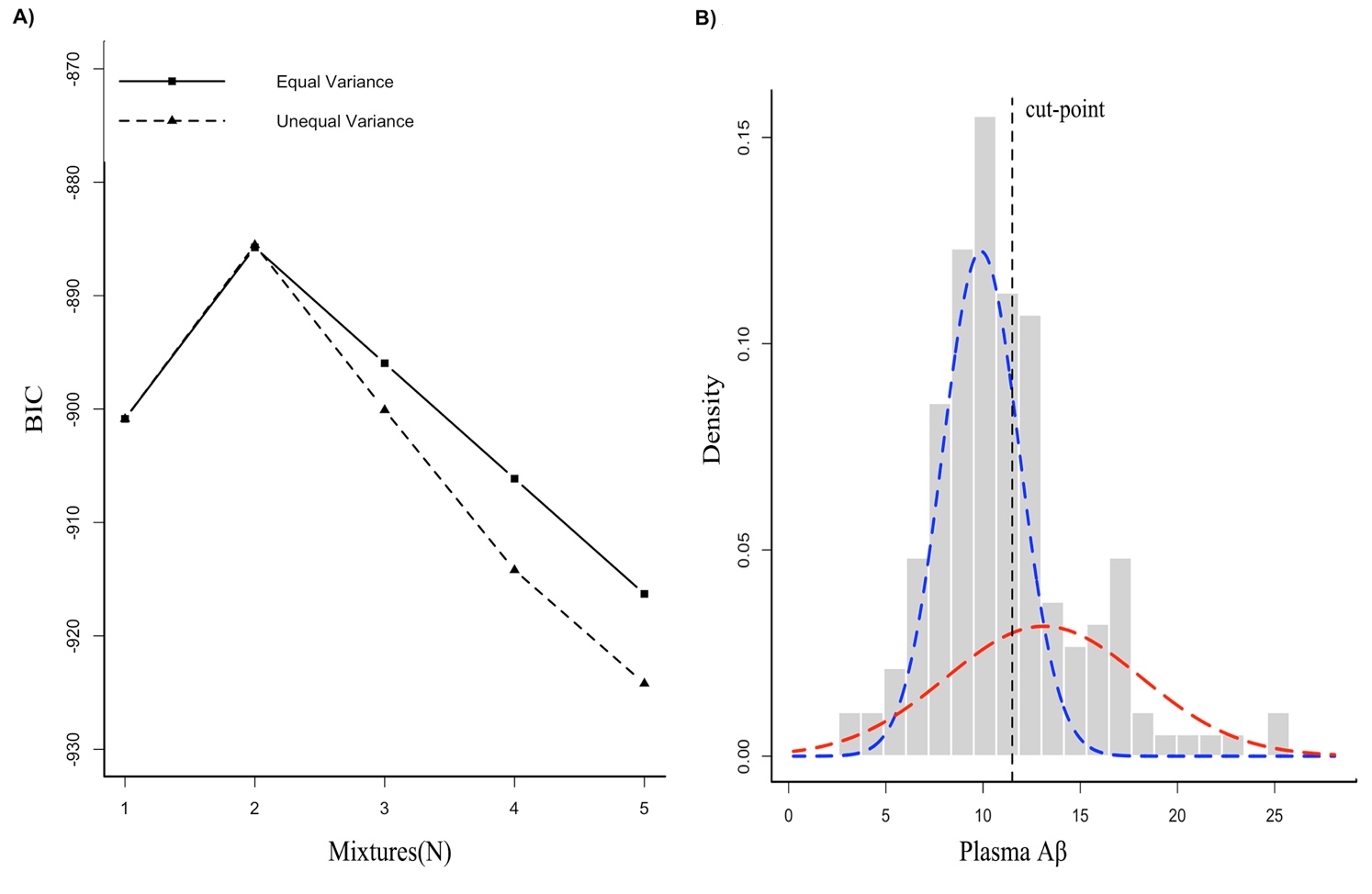


Figure e1 (A) Gaussian mixture models containing 1-5 mixtures (distributions) were fit to Aβ values in SILCODE. The BIC that evaluated which model is best is plotted on the y-axis (larger is better) and the number of distributions is on the x-axis. For SILCODE, a bimodal distribution best fitted the data. (B) Probability density functions for the estimated Gaussian distributions of the best-fit model are overlaid on the cohort’s density histogram for Aβ values. The blue curve represents the abnormal Aβ distribution whereas the red curve represents the normal Aβ distribution. The black dashed line represents the final cut-point.

# Supplementary Tables

Table e1 Demographic, genetic and neuroimaging factors associated with metrics of resilience to Aβ (including simultaneously resilience dependent measures)

|  | Cognitive resilience | | | Brain resilience | | Global resilience | | |  |
| --- | --- | --- | --- | --- | --- | --- | --- | --- | --- |
| Variables | Standardized β | P | Standardized β | | P | | Standardized β | P | |
| SILCODE |  |  |  | |  | |  |  | |
| Age | -0.0003 | 0.831 | -0.0926 | | **0.001** | | -0.0704 | **0.001** | |
| Sex | 0.0002 | 0.903 | -0.0796 | | 0.006 | | -0.0544 | 0.013 | |
| Educational level | -0.0019 | 0.19 | -0.0279 | | 0.282 | | -0.0166 | 0.434 | |
| APOE-ε4 | -0.0004 | 0.748 | -0.0117 | | 0.653 | | -0.0126 | 0.52 | |
| Whole brain volume | 0.0001 | 0.935 | -0.5546 | | **<0.001** | | -0.3984 | **<0.001** | |
| Right hippocampal volume | / | / | 0.6828 | | <0.001 | | 0.4838 | <0.001 | |
| Left hippocampal volume | / | / | 0.5786 | | <0.001 | | 0.4188 | <0.001 | |
| Memory composite score | 0.5894 | <0.001 | / | | / | | 0.3558 | <0.001 | |
| Execution composite score | 0.4334 | <0.001 | / | | / | | 0.2443 | <0.001 | |
| Language composite score | 0.3889 | <0.001 | / | | / | | 0.2191 | <0.001 | |
| ADNI |  |  |  | |  | |  |  | |
| Age | -0.0048 | 0.581 | -0.0601 | | **<0.001** | | -0.0468 | **<0.001** | |
| Sex | -0.0035 | 0.708 | -0.0567 | | <0.001 | | -0.0413 | <0.001 | |
| Educational level | -0.0107 | 0.203 | -0.0006 | | 0.959 | | -0.0064 | 0.54 | |
| APOE-ε4 | 0.0157 | 0.05 | 0.0066 | | 0.584 | | 0.014 | 0.161 | |
| Whole brain volume | 0.0092 | 0.318 | -0.1914 | | **<0.001** | | -0.1271 | **<0.001** | |
| Right hippocampal volume | / | / | 0.5815 | | <0.001 | | 0.409 | <0.001 | |
| Left hippocampal volume | / | / | 0.5327 | | <0.001 | | 0.3699 | <0.001 | |
| Memory composite score | 0.5129 | <0.001 | / | | / | | 0.3199 | <0.001 | |
| Execution composite score | 0.3883 | <0.001 | / | | / | | 0.2365 | <0.001 | |
| Language composite score | 0.4511 | <0.001 | / | | / | | 0.2794 | <0.001 | |

Table e2 Results of the mixed effect models to predict baseline and longitudinal cognitive decline in both cohorts (including simultaneously resilience dependent measures and their interactions of time)

| Main effects | SILCODE | | | ADNI | | |
| --- | --- | --- | --- | --- | --- | --- |
|  | β | t | P | β | t | P |
| CR | -16.926 | -1.226 | 0.222 | 0.733 | 0.288 | 0.773 |
| CR * years | 2.515 | 0.183 | 0.855 | 0.977 | 2.927 | **0.004** |
| BR | -0.186 | -0.459 | 0.647 | -1.275 | -1.048 | 0.296 |
| BR * years | -0.341 | -0.950 | 0.344 | 0.565 | 3.893 | **<0.001** |
| GR | -0.221 | -0.446 | 0.656 | -1.707 | -1.174 | 0.242 |
| GR * years | -0.358 | -0.746 | 0.457 | 0.420 | 2.100 | **0.036** |

Table e3 Information criteria for prediction models with and without resilience (based on MOCA and hippocampal volume)

| Model | AIC | △ | BIC | △ |
| --- | --- | --- | --- | --- |
| Cognitive predictor^a^ |  |  |  |  |
| without cognitive resilience | 187.59 | - | 193.68 | - |
| with cognitive resilience | 187.23 | -0.36 | 194.54 | 0.86 |
| Brain predictor^b^ |  |  |  |  |
| without brain resilience | 373.51 | - | 382.54 | - |
| with brain resilience | 368.88 | -4.64 | 379.71 | -2.83 |
| Combined^c^ |  |  |  |  |
| without global resilience | 187.59 | - | 194.91 | - |
| with global resilience | 186.20 | -1.40 | 194.73 | -0.18 |

Abbreviations: AIC, Akaike Information Criterion; BIC, Bayesian Information Criterion. All models were corrected for age, sex, educational level and APOE-ε4 status. ^a^ This model included MOCA as the predictor. ^b^ This model included hippocampal volume as the predictor. ^c^ This model included both MOCA and hippocampal volume as the predictor.
